# On Complex Matrix-Variate Dirichlet Averages and Its Applications in Various Sub-Domains

**DOI:** 10.3390/e25111534

**Published:** 2023-11-10

**Authors:** Princy Thankamani, Nicy Sebastian, Hans J. Haubold

**Affiliations:** 1 Department of Statistics, Cochin University of Science and Technology, Cochin 682 022, India; princyt@cusat.ac.in; 2 Department of Statistics, St. Thomas College Thrissur, Calicut University, Thenhipalam 680 001, India; nicycms@gmail.com; 3 Office for Outer Space Affairs, United Nations, Vienna International Center, A-1400 Vienna, Austria

**Keywords:** Dirichlet average, generalized type-1, type-2 Dirichlet measures, functions of matrix argument, Dirichlet measures in the complex domain, 15B52, 15B48, 26B10, 33C60, 33C65, 60E05, 62E15, 62H10, 62H05

## Abstract

This paper is about Dirichlet averages in the matrix-variate case or averages of functions over the Dirichlet measure in the complex domain. The classical power mean contains the harmonic mean, arithmetic mean and geometric mean (Hardy, Littlewood and Polya), which is generalized to the *y*-mean by de Finetti and hypergeometric mean by Carlson; see the references herein. Carlson’s hypergeometric mean averages a scalar function over a real scalar variable type-1 Dirichlet measure, which is known in the current literature as the Dirichlet average of that function. The idea is examined when there is a type-1 or type-2 Dirichlet density in the complex domain. Averages of several functions are computed in such Dirichlet densities in the complex domain. Dirichlet measures are defined when the matrices are Hermitian positive definite. Some applications are also discussed.

## 1. Introduction

Dirichlet averages are a type of weighted average used in mathematics and statistics. Given a function f(x) and a probability distribution p(x) defined over a domain *D*, the Dirichlet average of f(x) over *D* with respect to p(x) is defined as:

$${\langle f\rangle }_{p}=\frac{1}{\left|D\right|}\int_{D}f\left(x\right)p\left(x\right)dx,$$

where |D| is the measure of the domain *D*. Intuitively, the Dirichlet average is the average value of f(x) weighted by the probability distribution p(x) over the domain *D*. The name “Dirichlet average” comes from the fact that the formula for the average involves an integral that is similar to the Dirichlet integral, which is an important integral in the theory of functions of a complex variable. Dirichlet averages have connections to many other important mathematical concepts, such as harmonic analysis, the Fourier series, and the theory of functions of a complex variable. Dirichlet averages play an important role in various problems in number theory, including the study of prime numbers and the distribution of arithmetic functions; see [1,2], etc. Ref. [3] used Dirichlet averages in the study of random matrices. Dirichlet averages are used in the study of option pricing and risk management in finance; see [4,5] used it in the study of Bayesian inference and probabilistic modeling in machine learning. Dirichlet averages are used in the study of natural language processing and text analysis; see [6]. Overall, Dirichlet averages are an important mathematical tool that have many applications in various disciplines. They provide a way to compute the average value of a function over a probability distribution and have connections to many other important mathematical concepts.

In [7], there is a discussion of the classical power mean, which contains the harmonic, arithmetic and geometric means. The classical weighted average is of the following form:

$$f\left(b\right)={\left[w_{1}z_{1}^{b}+\ldots +w_{n}z_{n}^{b}\right]}^{\frac{1}{b}}.$$

where all the quantities are real scalar and where $w^{\prime }=\left(w_{1},\ldots ,w_{n}\right),z^{\prime }=\left(z_{1},\ldots ,z_{n}\right)$, $z_{j}>0,$$w_{j}>0,j=1,\ldots ,n$, and $\sum_{j=1}^{n}w_{j}=1$ with a prime denoting the transpose. For b=1, f(1) gives $\sum_{j=1}^{n}w_{j}z_{j}$ or the arithmetic mean; when b=−1, f(−1) provides ${\left[\sum_{j}\left(\frac{w_{j}}{z_{j}}\right)\right]}^{-1}=$ the harmonic mean and when $b\to 0_{+}$, then $f(0_{+})$ yields $\prod_{j=1}^{n}z_{j}^{w_{j}}=$ the geometric mean. This weighted mean f(b) is generalized to the *y*-mean by de Finetti [8] and to the hypergeometric mean by Carlson [9]. A real scalar variable type-1 Dirichlet measure is involved for the weights $\left(w_{1},\ldots ,w_{n-1}\right)$ in Carlson’s generalization, and then average of a given function is taken over this Dirichlet measure. In the current literature this is known as Dirichlet average of that function, the function need not reduce to the classical arithmetic, harmonic, and geometric means. Additionally, Carlson offered a comprehensive and in-depth examination of the many types of Dirichlet averages Carlson developed the notion of the Dirichlet average in his work, see also [9,10,11,12,13,14]. The integral mean of a function with respect to the Dirichlet measure is known as its “Drichlet average”.

The paper is organized as follows: Section 1 gives the basic concepts for developing the theory of the matrix-variate Dirichlet measure in complex domain. Dirichlet averages for a function of matrix argument in the complex domain are developed in Section 2. In Section 3, we discuss the complex matrix-variate type-2 Dirichlet measure and averages over some useful matrix-variate functions. The rectangular matrix-variate Dirichlet measure is presented in Section 4. In Section 5, we establish the connection between Dirichlet averages and Tsallis entropy. Section 6 provides an elaborate account of the diverse sub-domains in which the technology finds valuable applications.

### Complex Domain

In the present paper, we consider Dirichlet averages of various functions over Dirichlet measures in the complex domain in the matrix-variate cases. All matrices appearing in this paper are Hermitian positive definite and p×p unless stated otherwise. In order to distinguish, matrices in the complex domain will be denoted by a tilde as $\tilde{X}$ and real matrices will be written without the tilde as *X*. We consider real-valued scalar functions of the complex matrix argument and such functions will be averaged over a complex matrix-variate Dirichlet measure. The following standard notations will be used: $\det(\tilde{X})$ will mean the determinant of the complex matrix variable $\tilde{X}$. The absolute value of the determinant will be denoted by |det(·)|. This means that if $\det\left(\tilde{X}\right)=a+ib,i=\sqrt{-1}$ then $\sqrt{\left(a+ib)(a-ib\right)}={\left(a^{2}+b^{2}\right)}^{\frac{1}{2}}=\left|\det\left(\tilde{X}\right)\right|$. tr(·) will denote the trace of (·). $\int_{\tilde{X}}$ is integral over all $\tilde{X}$, where $\tilde{X}$ may be rectangular, square or positive definite. $\tilde{X}>O$ means that the p×p matrix $\tilde{X}$ is Hermitian positive definite. Constant matrices, whether real or in the complex domain, will be written without the tilde unless the fact is to be stressed, and in that case, we use a tilde. $O<A<\tilde{X}<B$ means $A>O,\tilde{X}-A>O,B-\tilde{X}>O$, where *A* and *B* are p×p constant positive definite matrices. Then,

$$\int_{O<A<\tilde{X}<B}f\left(\tilde{X}\right)d\tilde{X}=\int_{A}^{B}f\left(\tilde{X}\right)d\tilde{X},$$

means the integral over the Hermitian positive definite matrix $\tilde{X}>O$. When $O<A<\tilde{X}<B$ and $f(\tilde{X})$ is a real-valued scalar function of matrix argument, $\tilde{X}$ and $d\tilde{X}$ stand for the wedge product of differentials. Hence, for $\tilde{Z}=\left({\tilde{z}}_{jk}\right)=X+iY$, a m×n matrix of distinct variables ${\tilde{z}}_{jk}$’s, where *X* and *Y* are real matrices, $i=+\sqrt{-1}$. Then, the differential element $d\tilde{Z}=dX\wedge dY$, with dX and dY being the wedge products of differentials in *X* and *Y*, respectively. For example, $dX=\wedge_{j=1}^{m}\wedge_{k=1}^{n}dx_{jk}$ if $X=(x_{jk})$ and m×n. When $\tilde{Z}$ is Hermitian, then $X=X^{\prime }$ (symmetric) and $Y=-Y^{\prime }$ (skew symmetric), where prime denotes the transpose. In this case, $dX=\wedge_{j\geq k=1}^{p}dx_{jk}=\wedge_{j\leq k=1}^{p}dx_{jk}$ and $dY=\wedge_{j<k=1}^{p}dy_{jk}=\wedge_{j>k=1}^{p}dy_{jk}$. $\int_{\tilde{X}>O}f\left(\tilde{X}\right)d\tilde{X}$ means the integral over the Hermitian positive definite matrix $\tilde{X}>O$. It is a multivariate integral over all ${\tilde{x}}_{jk}$’s where $\tilde{X}=\left({\tilde{x}}_{jk}\right)$, ${\tilde{x}}_{jk}$’s are in the complex domain. The complex matrix-variate gamma function will be denoted by ${\tilde{\Gamma }}_{p}\left(\alpha \right)$, which has the following expression and integral representation:

(1) $${\tilde{\Gamma }}_{p}\left(\alpha \right)=\pi^{\frac{p(p-1)}{2}}\Gamma \left(\alpha \right)\Gamma \left(\alpha -1\right)\ldots \Gamma \left(\alpha -\left(p-1\right)\right),ℜ\left(\alpha \right)>p-1\;$$

and

(2) $${\tilde{\Gamma }}_{p}\left(\alpha \right)=\int_{\tilde{X}>O}{\left|\det\left(\tilde{X}\right)\right|}^{\alpha -p}e^{-\mathrm{tr}(\tilde{X})}d\tilde{X},ℜ\left(\alpha \right)>p-1\;$$

where ℜ(·) means the real part of (·) and the integration is over all Hermitian positive definite matrix $\tilde{X}$. For our computations to follow, we will need some Jacobians of transformations in the complex domain. These will be listed here without proofs. For the proofs and for other such Jacobians, see [15].

**Lemma 1.**  *Let $\tilde{X}$ and $\tilde{Y}$ be m×n with mn distinct complex variables as elements. Let A be m×m and B be n×n nonsingular constant matrices. Then*

(3) $$\tilde{Y}=A\tilde{X}B,\det\left(A\right)\neq 0,\det\left(B\right)\neq 0\Rightarrow d\tilde{Y}={\left[\det\left(A^{*}A\right)\right]}^{n}{\left[\det\left(B^{*}B\right)\right]}^{m}d\tilde{X}\;$$

where $A^{*}$ and $B^{*}$ denote the conjugate transposes of A and B, respectively; if X,Y,A,B are real then

(3a) $$Y=AXB\Rightarrow dY={\left[\det\left(A\right)\right]}^{n}{\left[\det\left(B\right)\right]}^{m}dX\;$$

*and if a is a scalar quantity then*

(3b) $$\tilde{Y}=a\tilde{X}\Rightarrow d\tilde{Y}={\left|a\right|}^{2mn}d\tilde{X}.\;$$

**Lemma 2.**  *Let $\tilde{X}$ be p×p and Hermitian matrix of distinct complex variables as elements, except for Hermitianness. Let A be a nonsingular constant matrix. Then*

(4) $$\tilde{Y}=A\tilde{X}A^{*}\Rightarrow d\tilde{Y}={\left|\det\left(A\right)\right|}^{-2p}d\tilde{X}.\;$$

*If $A,X,Y,X=X^{\prime }$ are real then*

(4a) $$Y=AXA^{\prime }\Rightarrow dY={\left[\det\left(A\right)\right]}^{p+1}dX.\;$$

*If $Y,X,a,X=X^{\prime }$ and a scalar, then*

(4b) $$Y=aX\to dY=a^{\frac{p(p+1)}{2}}dX\;$$

**Lemma 3.**  *Let $\tilde{X}$ be p×p and nonsingular with the regular inverse ${\tilde{X}}^{-1}$. Then*

(5) $$\tilde{Y}={\tilde{X}}^{-1}\Rightarrow d\tilde{Y}=\left\{\begin{array}{c} |\det\left({\tilde{X}}^{*}\tilde{X}\right){|}^{-2p}d\tilde{X}\;for\;a\;general\;\tilde{X}\\ |\det\left({\tilde{X}}^{*}\tilde{X}\right){|}^{-p}d\tilde{X}\;for\;\tilde{X}={\tilde{X}}^{*}\;or\;\tilde{X}=-{\tilde{X}}^{*} \end{array}\right.\;$$

**Lemma 4.**  *Let $\tilde{X}$ be p×p Hermitian positive definite of distinct elements, except for Hermitian positive definiteness. Let $\tilde{T}$ be a lower triangular matrix where $\tilde{T}=\left({\tilde{t}}_{jk}\right),{\tilde{t}}_{jk}=0,j<k,{\tilde{t}}_{jk},j\geq k$ are distinct, ${\tilde{t}}_{kk}=t_{kk}>0,k=1,\ldots ,p$, that is, the diagonal elements are real and positive. Then*

(6) $$\tilde{X}=\tilde{T}{\tilde{T}}^{*}\Rightarrow d\tilde{X}=2^{p}\left\{\prod_{k=1}^{p}t_{kk}^{2(p-k)+1}\right\}d\tilde{T}.\;$$

With the help of Lemma 4, we can evaluate the complex matrix-variate gamma integral in (2) and show that it is equal to the expression in (1). When Lemma 4 is applied to the integral in (2), the integral splits into *p* integrals of the form

$$\prod_{k=1}^{p}2\int_{0}^{\infty }{\left(t_{kk}^{2}\right)}^{\left(\alpha -p\right)+\frac{1}{2}\left(2\left(p-k\right)+1\right)}e^{-t_{kk}^{2}}dt_{kk}=\prod_{k=1}^{p}\Gamma \left(\alpha -\left(k-1\right)\right),ℜ\left(\alpha \right)>k-1,k=1,\ldots ,p$$

which results in the final condition as ℜ(α)>p−1, and p(p−1)/2 integrals of the form

$$\begin{array}{cc} \prod_{j>k}\int_{-\infty }^{\infty }e^{-|{\tilde{t}}_{jk}{|}^{2}}d{\tilde{t}}_{jk}\; & =\prod_{j>k}\int_{-\infty }^{\infty }\int_{-\infty }^{\infty }e^{-(t_{jk1}^{2}+t_{jk2}^{2})}dt_{jk1}\wedge dt_{jk2}\\ \; & =\prod_{j>k}\sqrt{\pi }\sqrt{\pi }=\pi^{\frac{p(p-1)}{2}},{\left|{\tilde{t}}_{jk}\right|}^{2}=t_{jk1}^{2}+t_{jk2}^{2}. \end{array}$$

Thus, the integral in (2) reduces to the expression in (1).

**Lemma 5.**  Let $\tilde{X}$ be n×p,n≥p matrix of full rank p. Let $\tilde{S}={\tilde{X}}^{*}\tilde{X}$, a p×p Hermitian positive definite matrix. Let $d\tilde{X}$ and $d\tilde{S}$ denote the wedge product of the differentials in $\tilde{X}$ and $\tilde{S}$, respectively. Then

(7) $$d\tilde{X}={\left|\det\left(\tilde{S}\right)\right|}^{n-p}\frac{\pi^{np}}{{\tilde{\Gamma }}_{p}\left(n\right)}d\tilde{S}.\;$$

This is a very important result because $\tilde{X}$ is a rectangular matrix with mn distinct elements, whereas $\tilde{S}$ is Hermitian positive definite and p×p. With the help of the above lemmas, we will average a few functions over the Dirichlet measures in the complex domain.

## 2. Dirichlet Averages for Functions of Matrix Argument in the Complex Domain

The Dirichlet distributions of real types 1 and 2 are generalized to standard distributions of beta and type-2. The literature contains these distributions, their characteristics, and a few generalizations in the form of Liouville distributions. Dirichlet type-1 and type-2 matrix-variate analogues can be found in the literature; [15] is one example. Generalizations of matrix variables to the Liouville family can be observed in [16]. Matrix-variate distributions, not generalized Dirichlet, may be seen from [17,18,19] provides examples of the use of scalar variable Dirichlet models in random division and other geometrical possibilities.

All the matrices appearing in this section are p×p Hermitian positive definite unless stated otherwise. Consider the following complex matrix-variate type-1 Dirichlet measure:

(8) $$\begin{array}{cc} f_{1}\left({\tilde{X}}_{1},\ldots ,{\tilde{X}}_{k}\right) & ={\tilde{D}}_{k}|\det\left({\tilde{X}}_{1}\right){|}^{\alpha_{1}-p}\ldots {\left|\det\left({\tilde{X}}_{k}\right)\right|}^{\alpha_{k}-p}\\ \; & \times |\det\left(I-{\tilde{X}}_{1}-\ldots -{\tilde{X}}_{k}\right){|}^{\alpha_{k+1}-p} \end{array}$$

where ${\tilde{X}}_{1},\ldots {\tilde{X}}_{k}$ are p×p Hermitian positive definite, that is, ${\tilde{X}}_{j}>O,j=1,\ldots ,k$, such that $I-{\tilde{X}}_{j}>O,j=1,\ldots ,k,I-\left({\tilde{X}}_{1}+\ldots +{\tilde{X}}_{k}\right)>O$. The normalizing constant ${\tilde{D}}_{k}$ can be evaluated by integrating out matrices one at a time and the individual integrals are evaluated by using a complex matrix-variate type-1 beta integral of the form

(9) $$\int_{O}^{I}|\det\left(\tilde{X}\right){|}^{\alpha -p}{\left|\det\left(I-\tilde{X}\right)\right|}^{\beta -p}d\tilde{X}=\frac{{\tilde{\Gamma }}_{p}\left(\alpha \right){\tilde{\Gamma }}_{p}\left(\beta \right)}{{\tilde{\Gamma }}_{p}\left(\alpha +\beta \right)},ℜ\left(\alpha \right)>p-1,ℜ\left(\beta \right)>p-1\;$$

where ${\tilde{\Gamma }}_{p}\left(\alpha \right)$ is given in (1). It can be shown that the normalizing constant is the following:

(10) $${\tilde{D}}_{k}=\frac{{\tilde{\Gamma }}_{p}\left(\alpha_{1}+\ldots +\alpha_{k+1}\right)}{{\tilde{\Gamma }}_{p}\left(\alpha_{1}\right)\ldots {\tilde{\Gamma }}_{p}\left(\alpha_{k+1}\right)}\;$$

for $ℜ(\alpha_{j})>p-1,j=1,\ldots ,k+1$. Since (10) is a probability measure, $f({\tilde{X}}_{1},\ldots ,{\tilde{X}}_{k})$ is non-negative for all ${\tilde{X}}_{j},j=1,\cdots ,k$ and the total integral is one. It is a Dirichlet measure associated with a Dirichlet density, and it is also a statistical density; hence, we can denote the averages of given functions as the expected values of those functions, denoted by E(·). Let us consider a few functions and take their averages over the complex matrix-variate Dirichlet measure in (8). Let

(11) $$\phi_{1}\left({\tilde{X}}_{1},\ldots ,{\tilde{X}}_{k}\right)=|\det\left({\tilde{X}}_{1}\right){|}^{\gamma_{1}}\ldots {\left|\det\left({\tilde{X}}_{k}\right)\right|}^{\gamma_{k}}.\;$$

Then, the average of (11) over the measure in (8) is given by

$$\begin{array}{cc} E[\phi_{1}] & ={\tilde{D}}_{k}\int_{{\tilde{X}}_{1},\ldots ,{\tilde{X}}_{k}}|\det\left({\tilde{X}}_{1}\right){|}^{\alpha_{1}+\gamma_{1}-p}\ldots {\left|\det\left({\tilde{X}}_{k}\right)\right|}^{\alpha_{k}+\gamma_{k}-p}\\ \; & \times |\det\left(I-{\tilde{X}}_{1}-\ldots -{\tilde{X}}_{k}\right){|}^{\alpha_{k+1}-p}d{\tilde{X}}_{1}\wedge \ldots .\wedge d{\tilde{X}}_{k}. \end{array}$$

Note that the only change is that $\alpha_{j}$ is changed to $\alpha_{j}+\gamma_{j}$ for j=1,…,k; hence, the result is available from the normalizing constant. That is,

(12) $$E\left[\phi_{1}\right]=\left\{\prod_{j=1}^{k}\frac{{\tilde{\Gamma }}_{p}\left(\alpha_{j}+\gamma_{j}\right)}{{\tilde{\Gamma }}_{p}\left(\alpha_{j}\right)}\right\}\frac{{\tilde{\Gamma }}_{p}\left(\alpha_{1}+\ldots +\alpha_{k}\right)}{{\tilde{\Gamma }}_{p}\left(\alpha_{1}+\gamma_{1}+\ldots +\alpha_{k}+\gamma_{k}+\alpha_{k+1}\right)},\;$$

for $ℜ\left(\alpha_{j}+\gamma_{j}\right)>p-1,j=1,\ldots ,k,ℜ\left(\alpha_{k+1}\right)>p-1$. Let

(13) $$\phi_{2}\left({\tilde{X}}_{1},\ldots ,{\tilde{X}}_{k}\right)={\left|\det\left(I-{\tilde{X}}_{1}-\ldots -{\tilde{X}}_{k}\right)\right|}^{\delta }.\;$$

Then, in the integral for $E[\phi_{2}]$ the only change is that the parameter $\alpha_{k+1}$ is changed to $\alpha_{k+1}+\delta$. Hence, the result is available from the normalizing constant ${\tilde{D}}_{k}$. That is,

(14) $$E\left[\phi_{2}\right]=\frac{{\tilde{\Gamma }}_{p}\left(\alpha_{k+1}+\delta \right)}{{\tilde{\Gamma }}_{p}\left(\alpha_{k+1}\right)}\frac{{\tilde{\Gamma }}_{p}\left(\alpha_{1}+\ldots +\alpha_{k+1}\right)}{{\tilde{\Gamma }}_{p}\left(\alpha_{1}+\ldots +\alpha_{k+1}+\delta \right)}\;$$

for $ℜ\left(\alpha_{k+1}+\delta \right)>p-1,ℜ\left(\alpha_{j}\right)>p-1,j=1,\ldots ,k$. The structure in (14) is also the structure of the δ-th moment of the determinant of the matrix with a complex matrix-variate type-1 beta distribution. Hence, this $\phi_{2}$ has an equivalent representation in terms of the determinant of a matrix with a complex matrix-variate type-1 beta distribution. Let

(15) $$\phi_{3}\left({\tilde{X}}_{1},\ldots ,{\tilde{X}}_{k}\right)=e^{\mathrm{tr}({\tilde{X}}_{1})}.\;$$

Let us evaluate the Dirichlet average for k=2. Then

$$\begin{array}{cc} E[\phi_{3}] & ={\tilde{D}}_{2}\int_{{\tilde{X}}_{1},{\tilde{X}}_{2}}e^{\mathrm{tr}({\tilde{X}}_{1})}|\det\left({\tilde{X}}_{1}\right){|}^{\alpha_{1}-p}{\left|\det\left({\tilde{X}}_{2}\right)\right|}^{\alpha_{2}-p}\\ \; & \times |\det\left(I-{\tilde{X}}_{1}-{\tilde{X}}_{2}\right){|}^{\alpha_{3}-p}d{\tilde{X}}_{1}\wedge \ldots \wedge d{\tilde{X}}_{3}. \end{array}$$

Take out $I-{\tilde{X}}_{1}$ from $\left|\det(I-{\tilde{X}}_{1}-{\tilde{X}}_{2})\right|$ and make the transformation

$${\tilde{U}}_{2}={\left(I-{\tilde{X}}_{1}\right)}^{-\frac{1}{2}}{\tilde{X}}_{2}{\left(I-{\tilde{X}}_{1}\right)}^{-\frac{1}{2}}.$$

Then, from Lemma 2, $d{\tilde{U}}_{2}={\left|\det\left(I-{\tilde{X}}_{1}\right)\right|}^{-p}d{\tilde{X}}_{2}$. Now, ${\tilde{U}}_{2}$ can be integrated out by using a complex matrix-variate type-1 beta integral given in (9). That is,

(16) $$\int_{O<{\tilde{U}}_{2}<I}|\det\left({\tilde{U}}_{2}\right){|}^{\alpha_{2}-p}{\left|\det\left(I-{\tilde{U}}_{2}\right)\right|}^{\alpha_{3}-p}d{\tilde{U}}_{2}=\frac{{\tilde{\Gamma }}_{p}\left(\alpha_{2}\right){\tilde{\Gamma }}_{p}\left(\alpha_{3}\right)}{{\tilde{\Gamma }}_{p}\left(\alpha_{2}+\alpha_{3}\right)}\;$$

for $ℜ\left(\alpha_{2}\right)>p-1,ℜ\left(\alpha_{3}\right)>p-1$. The ${\tilde{X}}_{1}$ integral to be evaluated is the following:

(17) $$\int_{{\tilde{X}}_{1}}e^{\mathrm{tr}({\tilde{X}}_{1})}|\det\left({\tilde{X}}_{1}\right){|}^{\alpha_{1}-p}{\left|\det\left(I-{\tilde{X}}_{1}\right)\right|}^{\alpha_{2}+\alpha_{3}-p}d\tilde{X_{1}}.\;$$

In order to evaluate the integral in (17), we can expand the exponential part by using zonal polynomials for complex argument; see [15,20]. We need a few notations and results from zonal polynomial expansions of determinants. The generalized Pochhammer symbol is the following:

(18) $${\left[a\right]}_{M}=\prod_{j=1}^{p}{\left(a-j+1\right)}_{k_{j}}=\frac{{\tilde{\Gamma }}_{p}\left(a,M\right)}{{\tilde{\Gamma }}_{p}\left(a\right)},{\tilde{\Gamma }}_{p}\left(a,M\right)={\tilde{\Gamma }}_{p}\left(a\right){\left[a\right]}_{M}\;$$

where the usual Pochhmmer symbol is

(19) $${\left(a\right)}_{m}=a\left(a+1\right)\ldots \left(a+m-1\right),a\neq 0,{\left(a\right)}_{0}=1\;$$

and *M* represents the partition, $M=\left(m_{1},\ldots ,m_{p}\right),m_{1}\geq m_{2}\geq \ldots \geq m_{p},m_{1}+\ldots +m_{p}=m$ and the zonal polynomial expansion for the exponential function is the following:

(20) $$e^{\mathrm{tr}(\tilde{X})}=\sum_{m=0}^{\infty }\sum_{M}\frac{{\tilde{C}}_{M}\left(\tilde{X}\right)}{m!}\;$$

where ${\tilde{C}}_{M}\left(\tilde{X}\right)$ is zonal polynomial of order *m* in the complex matrix argument $\tilde{X}$; see (6.1.18) of [15]. One result on zonal polynomial that we require will be stated here as a lemma.

**Lemma 6.** 
(21) $$\begin{array}{cc} \int_{O<\tilde{Z}<I} & |\det\left(\tilde{Z}\right){|}^{\alpha -p}{\left|\det\left(I-\tilde{Z}\right)\right|}^{\beta -p}{\tilde{C}}_{M}\left(\tilde{Z}\tilde{A}\right)d\tilde{Z}\\ \; & =\frac{{\tilde{\Gamma }}_{p}\left(\alpha ,M\right){\tilde{\Gamma }}_{p}\left(\beta \right)}{{\tilde{\Gamma }}_{p}\left(\alpha +\beta ,M\right)}{\tilde{C}}_{M}\left(\tilde{A}\right)\\ \; & =\frac{{\tilde{\Gamma }}_{p}\left(\alpha \right){\tilde{\Gamma }}_{p}\left(\beta \right)}{{\tilde{\Gamma }}_{p}\left(\alpha +\beta \right)}\frac{{\left(\alpha \right)}_{M}}{{\left(\alpha +\beta \right)}_{M}}{\tilde{C}}_{M}\left(\tilde{A}\right), \end{array}$$

*see also (6.1.21) of [15], for $ℜ\left(\alpha \right)>p-1,ℜ\left(\beta \right)>p-1,\tilde{A}>O$. By using (21), we can evaluate the ${\tilde{X}}_{1}$-integral in $E[\phi_{3}]$. That is,*

$$\begin{array}{cc} \int_{O<{\tilde{X}}_{1}<I} & e^{\mathrm{tr}(A{\tilde{X}}_{1})}|\det\left({\tilde{X}}_{1}\right){|}^{\alpha_{1}-p}{\left|\det\left(I-{\tilde{X}}_{1}\right)\right|}^{\alpha_{2}+\alpha_{3}-p}d{\tilde{X}}_{1}\\ \; & =\sum_{m=0}^{\infty }\sum_{M}\int_{O<{\tilde{X}}_{1}<I}\frac{{\tilde{C}}_{M}\left(\tilde{A}{\tilde{X}}_{1}\right)}{m!}|\det\left({\tilde{X}}_{1}\right){|}^{\alpha_{1}-p}{\left|\det\left(I-{\tilde{X}}_{1}\right)\right|}^{\alpha_{2}+\alpha_{3}-p}d{\tilde{X}}_{1}\\ \; & =\sum_{m=0}^{\infty }\sum_{M}\frac{{\tilde{C}}_{M}\left(\tilde{A}\right)}{m!}\frac{{\tilde{\Gamma }}_{p}\left(\alpha_{1},M\right){\tilde{\Gamma }}_{p}\left(\alpha_{2}+\alpha_{3}\right)}{{\tilde{\Gamma }}_{p}\left(\alpha_{1}+\alpha_{2}+\alpha_{3},M\right)}. \end{array}$$

*Now, with the result on ${\tilde{X}}_{2}$-integral, ${\tilde{D}}_{2}$ and the above result will result in all the gamma products being canceled and the final result is the following:*

(22) $$E\left[\phi_{3}\right]=\sum_{m=0}^{\infty }\sum_{M}\frac{{\tilde{C}}_{M}\left(\tilde{A}\right)}{m!}\frac{{\left(\alpha_{1}\right)}_{M}}{{\left(\alpha_{1}+\alpha_{2}+\alpha_{3}\right)}_{M}}={}_{1}F_{1}\left(\alpha_{1};\alpha_{1}+\alpha_{2}+\alpha_{3};\tilde{A}\right)\;$$

*for $ℜ(\alpha_{j})>p-1,j=1,2,3$ and ${}_{1}F_{1}$ is a confluent hypergeometric function of complex matrix argument $\tilde{A}$.*

## 3. Dirichlet Averages in Complex Matrix-Variate Type-2 Dirichlet Measure

Consider the type-2 Dirichlet measure

(23) $$\begin{array}{cc} f_{2}\left({\tilde{X}}_{1},\ldots ,{\tilde{X}}_{k}\right) & ={\tilde{D}}_{k}|\det\left({\tilde{X}}_{1}\right){|}^{\alpha_{1}-p}\ldots {\left|\det\left({\tilde{X}}_{k}\right)\right|}^{\alpha_{k}-p}\\ \; & \times |\det\left(I+{\tilde{X}}_{1}+\ldots +{\tilde{X}}_{k}\right){|}^{-(\alpha_{1}+\ldots +\alpha_{k+1})} \end{array}$$

for $ℜ(\alpha_{j})>p-1,j=1,\ldots ,k+1$ and it can be seen that the normalizing constant is the same as that in the type-1 Dirichlet measure. Let us evaluate some Dirichlet averages in the measure (23). Let

(24) $$\phi_{4}\left({\tilde{X}}_{1},\ldots ,{\tilde{X}}_{k}\right)=|\det\left({\tilde{X}}_{1}\right){|}^{\gamma_{1}}\ldots {\left|\det\left({\tilde{X}}_{k}\right)\right|}^{\gamma_{k}}.\;$$

Then, when the average is taken, the change is that $\alpha_{j}$ changes to $\alpha_{j}+\gamma_{j},j=1,\ldots ,k$; hence, one should be able to find the value from the normalizing constant by adjusting for $\alpha_{k+1}$. Write $\left(\alpha_{1}+\ldots +\alpha_{k+1}\right)=\left(\alpha_{1}+\gamma_{1}+\ldots +\alpha_{k}+\gamma_{k}\right)+\left(\alpha_{k+1}-\gamma_{1}-\ldots -\gamma_{k}\right)$. That is, replace $\alpha_{j}$ by $\alpha_{j}+\gamma_{j},j=1,\ldots ,k$ and replace $\alpha_{k+1}$ by $\alpha_{k+1}-\gamma_{1}-\ldots -\gamma_{k}$ to obtain the result from the normalizing constant. Therefore,

(25) $$E\left[\phi_{4}\right]=\left\{\prod_{j=1}^{k}\frac{{\tilde{\Gamma }}_{p}\left(\alpha_{j}+\gamma_{j}\right)}{{\tilde{\Gamma }}_{p}\left(\alpha_{j}\right)}\right\}\frac{{\tilde{\Gamma }}_{p}\left(\alpha_{k+1}-\gamma_{1}-\ldots -\gamma_{k}\right)}{{\tilde{\Gamma }}_{p}\left(\alpha_{k+1}\right)}\;$$

for $ℜ(\alpha_{j}+\gamma_{j})>p-1,j=1,\ldots ,k$ and $ℜ\left(\alpha_{k+1}-\gamma_{1}-\ldots -\gamma_{k}\right)>p-1,ℜ\left(\alpha_{k+1}\right)>p-1$. Thus, only a few moments will exist, interpreting $E[\phi_{4}]$ as the product moment of the determinants of ${\tilde{X}}_{1},\ldots {\tilde{X}}_{k}$. Let

(26) $$\phi_{5}\left({\tilde{X}}_{1},\ldots ,{\tilde{X}}_{k}\right)={\left|\det\left(I+{\tilde{X}}_{1}+\ldots +{\tilde{X}}_{k}\right)\right|}^{-\delta }.\;$$

Then, when the average is taken the only change in the integral is that $\alpha_{k+1}$ is changed to $\alpha_{k+1}+\delta$; hence, from the normalizing constant the result is the following:

(27) $$E\left[\phi_{5}\right]=\frac{{\tilde{\Gamma }}_{p}\left(\alpha_{k+1}+\delta \right)}{{\tilde{\Gamma }}_{p}\left(\alpha_{k+1}\right)}\frac{{\tilde{\Gamma }}_{p}\left(\alpha_{1}+\ldots +\alpha_{k+1}\right)}{{\tilde{\Gamma }}_{p}\left(\alpha_{1}+\ldots +\alpha_{k+1}+\delta \right)},\;$$

for $ℜ(\alpha_{k+1}+\delta )>p-1$, the other conditions on the parameters for ${\tilde{D}}_{k}$ remain the same. Observe that if ℜ(δ)>0, then the structure in (27) is that of the δ-th moment of the determinant of a complex matrix-variate type-1 beta matrix. Thus, this type-2 form gives a type-1 form result. Let

(28) $$\phi_{6}\left({\tilde{X}}_{1},{\tilde{X}}_{2}\right)=e^{-\mathrm{tr}(A{\tilde{X}}_{1})}{\left|\det\left(I+{\tilde{X}}_{1}\right)\right|}^{\alpha_{1}+\alpha_{3}}.\;$$

Then, the Dirichlet average of $\phi_{6}$ in the complex matrix-variate type-2 Dirichlet measure in (23) for k=2 is the following:

$$\begin{array}{cc} E[\phi_{6}] & ={\tilde{D}}_{2}\int_{{\tilde{X}}_{1},{\tilde{X}}_{2}}e^{-\mathrm{tr}({\tilde{X}}_{1})}|\det\left(I+{\tilde{X}}_{1}\right){|}^{\alpha_{2}+\alpha_{3}}|\det\left({\tilde{X}}_{1}\right){|}^{\alpha_{1}-p}{\left|\det\left({\tilde{X}}_{2}\right)\right|}^{\alpha_{2}-p}\\ \; & \times |\det\left(I+{\tilde{X}}_{1}+{\tilde{X}}_{2}\right){|}^{-(\alpha_{1}+\alpha_{2}+\alpha_{3})}d{\tilde{X}}_{1}\wedge \ldots d{\tilde{X}}_{3}. \end{array}$$

Take out $\left(I+{\tilde{X}}_{1}\right)$ from $I+{\tilde{X}}_{1}+{\tilde{X}}_{2}$ and make the transformation

$${\tilde{U}}_{2}={\left(I+{\tilde{X}}_{1}\right)}^{-\frac{1}{2}}{\tilde{X}}_{2}{\left(I+{\tilde{X}}_{1}\right)}^{-\frac{1}{2}}\Rightarrow d{\tilde{U}}_{2}={\left|\det\left(I+{\tilde{X}}_{1}\right)\right|}^{-p}d{\tilde{X}}_{2}.$$

The $\tilde{U_{2}}$-integral gives

(29) $$\int_{{\tilde{U}}_{2}>O}|\det\left({\tilde{U}}_{2}\right){|}^{\alpha_{2}-p}{\left|\det\left(I+{\tilde{U}}_{2}\right)\right|}^{-(\alpha_{1}+\alpha_{2}+\alpha_{3})}d{\tilde{U}}_{2}=\frac{{\tilde{\Gamma }}_{p}\left(\alpha_{2}\right){\tilde{\Gamma }}_{p}\left(\alpha_{1}+\alpha_{3}\right)}{{\tilde{\Gamma }}_{p}\left(\alpha_{1}+\alpha_{2}+\alpha_{3}\right)}.\;$$

Observe that the exponent becomes zero and the factor containing $\left|\det(I+{\tilde{X}}_{1})\right|$ disappears. Then, the ${\tilde{X}}_{1}$-integral is

(30) $$\int_{{\tilde{X}}_{1}>O}|\det\left({\tilde{X}}_{1}\right){|}^{\alpha_{1}-p}e^{-\mathrm{tr}(A{\tilde{X}}_{1})}d{\tilde{X}}_{1}={\tilde{\Gamma }}_{p}\left(\alpha_{1}\right){\left|\det\left(A\right)\right|}^{-\alpha_{1}}.\;$$

The results from (29), (30) and ${\tilde{D}}_{2}$ gives the final result as follows:

(31) $$E\left[\phi_{6}\right]=\frac{{\tilde{\Gamma }}_{p}\left(\alpha_{1}+\alpha_{3}\right)}{{\tilde{\Gamma }}_{p}\left(\alpha_{3}\right)}{\left|\det\left(A\right)\right|}^{-\alpha_{1}}\;$$

and the original conditions on the parameters remain the same and no further conditions are needed, where A>O. Note that if $\phi_{6}$ did not have the factor $|\det\left(I+{\tilde{X}}_{1}\right){|}^{\alpha_{1}+\alpha_{3}}$, a factor containing $\left|\det(I+{\tilde{X}}_{1})\right|$ would also have been present, then the ${\tilde{X}}_{1}$-integral would have gone in terms of a Whittaker function of matrix argument; see [15].

## 4. Dirichlet Averages in Complex Rectangular Matrix-Variate Dirichlet Measure

Let $B_{j}$ be $n_{j}\times n_{j}$ a Hermitian positive definite constant matrix and let $B_{j}^{\frac{1}{2}}$ denote the Hermitian positive definite square root of $B_{j}$. Let ${\tilde{X}}_{j}$ be a $n_{j}\times p$, $n_{j}\geq p$ matrix of full rank *p* so that ${\tilde{X}}_{j}^{*}{\tilde{X}}_{j}={\tilde{S}}_{j}>O$ or ${\tilde{S}}_{j}$ is a Hermitian positive definite. Observe that for p=1, ${\tilde{X}}_{j}^{*}B_{j}{\tilde{X}}_{j}$ is a positive definite Hermitian form. Hence, our results to follow will also cover results on Hermitian forms. Consider the model

(32) $$\begin{array}{cc} f_{3}\left({\tilde{X}}_{1},\ldots ,{\tilde{X}}_{k}\right) & ={\tilde{G}}_{k}|\det\left({\tilde{X}}_{1}^{*}B_{1}{\tilde{X}}_{1}\right){|}^{\alpha_{1}}\ldots {\left|\det\left({\tilde{X}}_{k}^{*}B_{k}{\tilde{X}}_{k}\right)\right|}^{\alpha_{k}}\\ \; & \times |\det\left(I-{\tilde{X}}_{1}^{*}B_{1}{\tilde{X}}_{1}-\ldots -{\tilde{X}}_{k}^{*}B_{k}{\tilde{X}}_{k}\right){|}^{\alpha_{k+1}-p} \end{array}$$

where ${\tilde{G}}_{k}$ is the normalizing constant and $O<{\tilde{X}}_{j}^{*}B_{j}{\tilde{X}}_{j}<I,j=1,\ldots ,k,O<{\tilde{X}}_{1}^{*}B_{1}{\tilde{X}}_{1}+\ldots +{\tilde{X}}_{k}^{*}B_{k}{\tilde{X}}_{k}<I,j=1,\ldots ,k$. The normalizing constant is evaluated by using the following procedure. Let ${\tilde{Y}}_{j}=B_{j}^{\frac{1}{2}}{\tilde{X}}_{j}\Rightarrow d\tilde{Y_{j}}={\left|\det\left(B_{j}\right)\right|}^{p}d{\tilde{X}}_{j}$ from Lemma 1. Let ${\tilde{Y}}_{j}^{*}{\tilde{Y}}_{j}={\tilde{S}}_{j}$. Then, from Lemma 5 we have

(33) $$d{\tilde{Y}}_{j}=\frac{\pi^{n_{j}p}}{{\tilde{\Gamma }}_{p}\left(n_{j}\right)}{\left|\det\left({\tilde{S}}_{j}\right)\right|}^{n_{j}-p}d{\tilde{X}}_{j}.\;$$

Then,

(34) $$d{\tilde{X}}_{1}\wedge \ldots \wedge d{\tilde{X}}_{k}=\{\prod_{j=1}^{k}\frac{\pi^{n_{j}p}}{{\tilde{\Gamma }}_{p}\left(n_{j}\right)}|\det\left({\tilde{B}}_{j}\right){|}^{-p}|\det\left({\tilde{S}}_{j}\right){|}^{n_{j}-p}\}d{\tilde{S}}_{1}\wedge \ldots \wedge d{\tilde{S}}_{k}.\;$$

Since the total integral is 1, we have

$$\begin{array}{cc} 1 & =\int_{{\tilde{X}}_{1},\ldots {\tilde{X}}_{k}}f_{3}\left({\tilde{X}}_{1},\ldots ,{\tilde{X}}_{k}\right)d{\tilde{X}}_{1}\wedge \ldots \wedge d{\tilde{X}}_{k}\\ \; & ={\tilde{G}}_{k}\left\{\prod_{j=1}^{k}\frac{\pi^{n_{j}p}}{{\tilde{\Gamma }}_{p}\left(n_{j}\right)}\right\}\int_{{\tilde{S}}_{1},\ldots ,{\tilde{S}}_{k}}{\left|\det\left({\tilde{S}}_{1}\right)\right|}^{\alpha_{1}+n_{1}-p}\ldots \\ \; & \times |\det\left({\tilde{S}}_{k}\right){|}^{\alpha_{k}+n_{k}-p}{\left|\det\left(I-{\tilde{S}}_{1}-\ldots -{\tilde{S}}_{k}\right)\right|}^{\alpha_{k+1}-p}d{\tilde{S}}_{1}\wedge \ldots \wedge d{\tilde{S}}_{k}. \end{array}$$

Now, evaluating the type-1 Dirichlet integrals over the ${\tilde{S}}_{j}$’s, one obtains the following result:

(35) $$\begin{array}{cc} {\tilde{G}}_{k} & =\{\prod_{j=1}^{k}|\det\left(B_{j}\right){|}^{p}\frac{{\tilde{\Gamma }}_{p}\left(n_{j}\right)}{\pi^{n_{j}p}}\frac{1}{{\tilde{\Gamma }}_{p}\left(\alpha_{j}+n_{j}\right)}\}\\ \; & \times \frac{{\tilde{\Gamma }}_{p}\left(\alpha_{1}+\ldots +\alpha_{k+1}+n_{1}+\ldots +n_{k}\right)}{{\tilde{\Gamma }}_{p}\left(\alpha_{k+1}\right)} \end{array}$$

for $B_{j}>O,ℜ\left(\alpha_{j}+n_{j}\right)>p-1,j=1,\ldots ,k,ℜ\left(\alpha_{k+1}\right)>p-1$. Thus, (32) with (35) defines a rectangular complex matrix-variate type-1 Dirichlet measure. There is a corresponding type-2 Dirichlet measure, given by the following:

(36) $$\begin{array}{cc} f_{4}\left({\tilde{X}}_{1},\ldots ,{\tilde{X}}_{k}\right) & ={\tilde{G}}_{k}|\det\left({\tilde{X}}_{1}\right){|}^{\alpha_{1}}\ldots {\left|\det\left({\tilde{X}}_{k}\right)\right|}^{\alpha_{k}}\\ \; & \times |\det\left(I+{\tilde{X}}_{1}+\ldots +{\tilde{X}}_{k}\right){|}^{-(\alpha_{1}+\ldots +\alpha_{k+1}+n_{1}+\ldots +n_{k})} \end{array}$$

for $B_{j}>O,ℜ\left(\alpha_{j}+n_{j}\right)>p-1,j=1,\ldots ,k,ℜ\left(\alpha_{k+1}\right)>p-1$ and ${\tilde{G}}_{k}$ is the same as the one appearing in (35). Let us compute the Dirichlet averages of some functions in the type-2 rectangular complex matrix-variate Dirichlet measure in (36). Let

(37) $$\phi_{7}\left({\tilde{X}}_{1},\ldots ,{\tilde{X}}_{k}\right)=|\det\left({\tilde{X}}_{1}\right){|}^{\gamma_{1}}\ldots {\left|\det\left({\tilde{X}}_{k}\right)\right|}^{\gamma_{k}}.\;$$

Then, when we take the expected value of $\phi_{7}$ in (36) the only change is that $\alpha_{j}$ changes to $\alpha_{j}+\gamma_{j},j=1,\ldots ,k$; hence, the final result is available from the normalizing constant. Therefore

(38) $$E\left[\phi_{7}\right]=\left\{\prod_{j=1}^{k}\frac{{\tilde{\Gamma }}_{p}\left(\alpha_{j}+n_{j}+\gamma_{j}\right)}{{\tilde{\Gamma }}_{p}\left(\alpha_{j}+n_{j}\right)}\right\}\frac{{\tilde{\Gamma }}_{p}\left(\alpha_{k+1}-\gamma_{1}-\ldots -\gamma_{k}\right)}{{\tilde{\Gamma }}_{p}\left(\alpha_{k+1}\right)}\;$$

for $ℜ\left(\alpha_{j}+n_{j}+\gamma_{j}\right)>p-1,j=1,\ldots ,k,ℜ\left(\alpha_{k+1}-\gamma_{1}-\ldots -\gamma_{k}\right)>p-1,ℜ\left(\alpha_{k+1}\right)>p-1$. Let

(39) $$\phi_{8}\left({\tilde{X}}_{1},\ldots ,{\tilde{X}}_{k}\right)={\left|\det\left(I+{\tilde{X}}_{1}+\ldots +{\tilde{X}}_{k}\right)\right|}^{-\delta }.\;$$

Then, the only change is that $\alpha_{k+1}$ goes to $\alpha_{k+1}+\delta$ in the integral and no other change is there; hence, the average is available from the normalizing constant. That is,

(40) $$E\left[\phi_{8}\right]=\frac{{\tilde{\Gamma }}_{p}\left(\alpha_{k+1}+\delta \right)}{{\tilde{\Gamma }}_{p}\left(\alpha_{k+1}\right)}\frac{{\tilde{\Gamma }}_{p}\left(\alpha_{1}+\ldots +\alpha_{k+1}+n_{1}+\ldots +n_{k}\right)}{{\tilde{\Gamma }}_{p}\left(\alpha_{1}+\ldots +\alpha_{k+1}+n_{1}+\ldots +n_{k}+\delta \right)}\;$$

for $ℜ\left(\alpha_{j}+n_{j}\right)>p-1,j=1,\ldots ,k,ℜ\left(\alpha_{k+1}+\delta \right)>p-1,ℜ\left(\alpha_{k+1}\right)>p-1$.

The case p=1 in the complex rectangular matrix-variate type-1 Dirichlet measure is very interesting. We have a set of Hermitian positive definite quadratic forms here having a joint density of the following form:

(41) $$\begin{array}{cc} f_{5}\left({\tilde{X}}_{1},\ldots ,{\tilde{X}}_{k}\right) & ={\tilde{G}}_{k}{\left[{\tilde{X}}_{1}^{*}B_{1}{\tilde{X}}_{1}\right]}^{\alpha_{1}}\ldots {\left[{\tilde{X}}_{k}^{*}B_{k}{\tilde{X}}_{k}\right]}^{\alpha_{k}}\\ \; & \times |\det\left(I-\left[{\tilde{X}}_{1}^{*}B_{1}{\tilde{X}}_{1}\right]-\ldots -\left[{\tilde{X}}_{k}^{*}B_{k}{\tilde{X}}_{k}\right]\right){|}^{\alpha_{k+1}-p} \end{array}$$

where $B_{j}>O$, and ${\tilde{X}}_{j}^{*}B_{j}{\tilde{X}}_{j}$ is a scalar quantity, j=1,…,k. Consider the same types of transformations as before. ${\tilde{Y}}_{j}=B_{j}^{\frac{1}{2}}{\tilde{X}}_{j}$. Then, ${\tilde{Y}}_{j}^{*}{\tilde{Y}}_{j}=|{\tilde{y}}_{j1}{|}^{2}+\ldots +{\left|{\tilde{y}}_{jn_{j}}\right|}^{2}$ or the sum or squares of the absolute values of ${\tilde{y}}_{jr}$ where ${\tilde{Y}}_{j}^{*}=\left({\tilde{y}}_{j1}^{*},\ldots ,{\tilde{y}}_{jn_{j}}^{*}\right)$. This is an isotropic point in in the $2n_{j}$-dimensional Euclidean space. From here, one can establish various connections to geometrical probability problems; see [19]. Also, (41) is associated with the theory of generalized Hermitian forms in pathway models; see [21]. Let us evaluate the *h*-th moment of

(42) $$\phi_{9}\left({\tilde{X}}_{1},\ldots ,{\tilde{X}}_{k}\right)={\left[{\tilde{X}}_{1}^{*}B_{1}{\tilde{X}}_{1}+\ldots +{\tilde{X}}_{k}^{*}B_{k}{\tilde{X}}_{k}\right]}^{h}\;$$

for p=1. For p>1 we have seen that this is not available directly but moments of $\left|\det(I-{\tilde{X}}_{1}^{*}B_{1}{\tilde{X}}_{1}-\ldots -{\tilde{X}}_{k}^{*}B_{k}{\tilde{X}}_{k})\right|$ was available. But for p=1, one can obtain the *h*-th moment of both for an arbitrary *h*. By computing the *h*-th moment of $\left[1-{\tilde{X}}_{1}^{*}B_{1}{\tilde{X}}_{1}-\ldots -{\tilde{X}}_{k}^{*}B_{k}{\tilde{X}}_{k}\right]$, for p=1, we note that for arbitrary *h*, this quantity and its complementary part $\left[{\tilde{X}}_{1}^{*}B_{1}{\tilde{X}}_{1}+\ldots +{\tilde{X}}_{k}^{*}B_{k}{\tilde{X}}_{k}\right]$ are both scalar variable type-1 beta distributed with the parameters $\left(\alpha_{k+1},\sum_{j=1}^{k}\left(\alpha_{j}+n_{j}\right)\right)$ and $\left(\sum_{j=1}^{k}\left(\alpha_{j}+n_{j}\right),\alpha_{k+1}\right)$, respectively. Then,

(43) $$E\left[\phi_{9}\right]=\frac{{\tilde{\Gamma }}_{p}\left(\sum_{j=1}^{k}\left(\alpha_{j}+n_{j}\right)+h\right)}{{\tilde{\Gamma }}_{p}\left(\sum_{j=1}^{k}\left(\alpha_{j}+n_{j}\right)\right)}\frac{{\tilde{\Gamma }}_{p}\left(\sum_{j=1}^{k}\left(\alpha_{j}+n_{j}\right)+\alpha_{k+1}\right)}{{\tilde{\Gamma }}_{p}\left(\sum_{j=1}^{k}\left(\alpha_{j}+n_{j}\right)+\alpha_{k+1}+h\right)}\;$$

for $ℜ\left(\alpha_{j}\right)>p-1,j=1,\ldots ,k+1,ℜ\left(\sum_{j=1}^{k}\left(\alpha_{j}+n_{j}\right)+h\right)>p-1$. Consider $\phi_{9}$ in the complex matrix-variate type-2 Dirichlet measure for p=1. Then, the *h*-th moment will reduce to the following:

(44) $$E\left[\phi_{9}\right]=\frac{{\tilde{\Gamma }}_{p}\left(\sum_{j=1}^{k}\left(\alpha_{j}+n_{j}\right)+h\right)}{{\tilde{\Gamma }}_{p}\left(\sum_{j=1}^{k}\left(\alpha_{j}+n_{j}\right)\right)}\frac{{\tilde{\Gamma }}_{p}\left(\alpha_{k+1}-h\right)}{{\tilde{\Gamma }}_{p}\left(\alpha_{k+1}\right)}\;$$

for $ℜ\left(\alpha_{k+1}-h\right)>p-1,ℜ\left(\alpha_{j}\right)>p-1,j=1,\ldots ,k+1,ℜ\left(\sum_{j=1}^{k}\left(\alpha_{j}+n_{j}\right)+h\right)>p-1$. 

Many such results can be obtained for the type-1 and type-2 Dirichlet measures in Hermitian positive definite Dirichlet measures or in rectangular matrix-variate Dirichlet measures.

## 5. A Connection to Tsallis Statistics of Non-Extensive Statistical Mechanics

Ref. [22] introduced an entropy measure and, by optimizing this entropy in an escort density, and under the constraint that the first moment in the escort density is prefixed which will correspond to a physical law of conservation of energy, obtained the famous Tsallis statistics of non-extensive statistical mechanics. Tsallis entropy is a variant of Havrda–Charvát entropy; see [23]. Havrda–Charvát entropy is an α-generalized Shannon entropy and Shannon entropy in the discrete distribution is the following:

(45) $$S\left(f\right)=-C\sum_{j=1}^{k}p_{j}\ln p_{j},p_{j}>0,j=1,\ldots ,k,p_{1}+\ldots +p_{k}=1\;$$

and its continuous version is the following:

(46) $$S\left(f\right)=-C\int_{x}f\left(x\right)\ln f\left(x\right)dx,f\left(x\right)\geq 0\mathrm{for}\;\mathrm{all}\;x\;and\;\int_{x}f\left(x\right)dx=1\;$$

where *C* is a constant. A generalized entropy, introduced by Mathai, is a variant of Havrda–Charvát entropy and Tsallis entropy in the real scalar variable case, but Mathai’s entropy is set in a very general framework. It is the following:

(47) $$M_{\alpha }\left(f\right)=\frac{\int_{X}{\left[f\left(X\right)\right]}^{1+\frac{a-\alpha }{\eta }}dX-1}{\alpha -a},\alpha \neq a,\eta >0\;$$

where *a* is a fixed anchoring point, α is the parameter of interest, η>0 is a fixed scaling factor or unit of measurement, f(X) is a real-valued scalar function of *X* such that f(X)≥0 for all *X*, $\int_{X}f\left(X\right)dX=1$ or f(X) is a statistical density where *X* can be a scalar or vector or matrix or a collection of matrices in the real or complex domain and dX is the wedge product of all distinct real scalar variables in *X*. For example, if $X^{\prime }=\left[x_{1},\ldots ,x_{p}\right]$, where $x_{j},j=1,\ldots ,p$ are distinct real scalar variables and a prime denoting the transpose, then $dX=dx_{1}\wedge \ldots \wedge dx_{p}=dX^{\prime }$. For two real scalar variables *x* and *y*, the wedge product of differentials is defined as dx∧dy=−dy∧dx, so that dx∧dx=0,dy∧dy=0. If $X=(x_{ij})$ is a p×q matrix of distinct real scalar variables $x_{ij}$’s, then $dX=\wedge_{i=1}^{p}\wedge_{j=1}^{q}dx_{ij}$. If $\tilde{X}=X_{1}+iX_{2},i=\sqrt{\left(-1\right)},X_{1},X_{2}$ is real, then $d\tilde{X}=dX_{1}\wedge dX_{2}$. If $X=[X_{1},\ldots ,X_{k}]$, a collection of matrices in the real domain, then $dX=dX_{1}\wedge \ldots \wedge dX_{k}$. If $\tilde{X}=\left[{\tilde{X}}_{1},\ldots ,{\tilde{X}}_{k}\right]$, then $d\tilde{X}=d{\tilde{X}}_{1}\wedge \ldots \wedge d{\tilde{X}}_{k}$. Thus, (47) is the expected value of ${\left[f\left(X\right)\right]}^{\frac{a-\alpha }{\eta }}$, where the deviation of α from the anchoring point *a* is measured in terms of η units.

When α→a in the real scalar case, we can see that (47) goes to Shannon’s entropy of (46). But (47) is set up in a very general framework. Let us consider (47) when *X* is a p×1 vector of distinct real scalar positive variables, $x_{j}>0,j=1,\ldots ,p$ and let $x_{1}+\ldots +x_{p}<1$ so that the $x_{j}$’s are in a unit ball. Let us optimize (47) under two product moment type constraints. Let

$$A=E{\left[x_{1}^{\alpha_{1}-1}\ldots x_{p}^{\alpha_{p}-1}\right]}^{\frac{a-\alpha }{\eta }}\mathrm{and}\;B=E\left[{\left(x_{1}^{\alpha_{1}-1}\ldots x_{p}^{\alpha_{p}-1}\right)}^{\frac{a-\alpha }{\eta }}\left(\sum_{j=1}^{p}x_{j}\right)\right]$$

for $ℜ(\alpha_{j})>0,j=1,\ldots ,p$, where ℜ(·) denotes the real part of (·). Let the constraints be *A* is prefixed and *B* is prefixed. If we use calculus of variation to optimize (47) under the above constraints, then the Euler equation is the following, where $\lambda_{1}$ and $\lambda_{2}$ are Lagrangian multipliers:

$$\frac{\partial }{\partial f}\left\{f^{1+\frac{a-\alpha }{\eta }}-\lambda_{1}{\left(x_{1}^{\alpha_{1}-1}\ldots x_{p}^{\alpha_{p}-1}\right)}^{\frac{a-\alpha }{\eta }}f-\lambda_{2}{\left(x_{1}^{\alpha_{1}-1}\ldots x_{p}^{\alpha_{p}-1}\right)}^{\frac{a-\alpha }{\eta }}\left(\sum_{j=1}^{p}x_{j}\right)f\right\}=0\Rightarrow$$

$$\begin{array}{cc} \left(1+\frac{a-\alpha }{\eta }\right)f^{\frac{a-\alpha }{\eta }} & =\lambda_{1}{\left(x_{1}^{\alpha_{1}-1}\ldots x_{p}^{\alpha_{p}-1}\right)}^{\frac{a-\alpha }{\eta }}+\lambda_{2}{\left(x_{1}^{\alpha_{1}-1}\ldots x_{p}^{\alpha_{p}-1}\right)}^{\frac{a-\alpha }{\eta }}\left(\sum_{j=1}^{p}x_{j}\right)\Rightarrow \\ f & =\lambda_{3}x_{1}^{\alpha_{1}-1}\ldots x_{p}^{\alpha_{p}-1}{\left[1+\lambda_{4}\sum_{j=1}^{p}x_{j}\right]}^{\frac{\eta }{a-\alpha }} \end{array}$$

for some $\lambda_{3}$ and $\lambda_{4}$. Let α<a. Then, let us take $\lambda_{4}=-b\left(a-\alpha \right),b>0$ so that the right side of the above equation for *f* can form a density with $\lambda_{3}$ being the normalizing constant there. If $\lambda_{4}=b\left(a-\alpha \right)$ with b>0,α<a, then the right side of *f* will be a positive exponential function and will not produce a density. Then,

(48) $$f=\lambda_{3}x_{1}^{\alpha_{1}-1}\ldots x_{p}^{\alpha_{p}-1}{\left[1-b\left(a-\alpha \right)\left(x_{1}+\ldots +x_{p}\right)\right]}^{\frac{\eta }{a-\alpha }},\eta >0,b>0,\alpha <a\;$$

is a Mathai’s pathway form of real scalar type-1 Dirichlet density. When α>a, then (48) switches into a real scalar type-2 Dirichlet density with the corresponding normalizing constant.

Note that for q=1, a q×q Hermitian positive definite matrix is a real scalar positive variable. Hence, (48) holds in the real and complex cases for q=1 of q×q real positive definite or Hermitian positive definite matrices $X_{1},\ldots ,X_{p}$ or ${\tilde{X}}_{1},\ldots ,{\tilde{X}}_{p}$. The above is an example of the connection of type-1 and type-2 Dirichlet models to Tsallis entropy.

## 6. Applications

For our applications in the theory of special functions, fractional calculus, mechanics, biology, probability, and stochastic processes, Dirichlet averages and their diverse approaches are used. In this section, the main areas where the applications of Dirichlet averages are presented:

### 6.1. Special Functions

Dirichlet averages were introduced by Carlson in his 1977 work. Carlson [10,11,12,13] observed that the straightforward idea of this kind of averaging generalizes and unifies a wide range of special functions, including various orthogonal polynomials and generalized hyper-geometric functions. The relationship between Dirichlet splines and an important class of hypergeometric functions of several variables is given in [14,24]. Numerous investigations of B-splines, including those by [14,25,26], used Dirichlet averages.

### 6.2. Fractional Calculus

The Dirichlet average of elementary functions like power function, exponential function, etc. is given by many notable mathematicians. There are many results available in the literature converting the elementary function into the summation form after taking the Dirichlet average of those functions, using the fractional integral, and obtaining new results; see [27,28,29,30,31,32]. Those results will be used in the future by mathematicians and scientists in a variety of fields.

### 6.3. Statistical Mechanics

Statistical mechanics is a branch of physics that studies the behaviour of large systems of particles, such as gases, liquids, and solids. In statistical mechanics, entropy is a measure of the degree of disorder or randomness in a system; for more details, see [33,34]. The greater the entropy, the more disordered the system. Dirichlet averages and statistical mechanics are connected through the concept of entropy. Dirichlet averages are a type of mathematical average that weighs a set of values according to a given probability distribution. For example, given a set of values $x_{1},x_{2},\ldots ,x_{n}$ and a probability distribution $p_{1},p_{2},\ldots ,p_{n},$ the Dirichlet average is defined as:

$$D\left(p,x\right)=\sum_{i=1}^{n}p_{i}x_{i}.$$

Statistical mechanics is a branch of physics that studies the behavior of large systems of particles, such as gases, liquids, and solids. The connection between Dirichlet averages and statistical mechanics comes from the fact that the Dirichlet average can be seen as a type of average energy of a system weighted by a probability distribution. In statistical mechanics, the average energy of a system is also weighted by a probability distribution, and the entropy of the system is related to the probability distribution of the energy states. In particular, the Boltzmann entropy of a system is given by:

$$S=-k\sum_{i=1}^{n}p_{i}\ln p_{i},$$

where *k* is the Boltzmann constant. This formula shows that the entropy of a system is proportional to the negative logarithm of the probability distribution of the energy states. Thus, Dirichlet averages and statistical mechanics are connected through the concept of entropy, which relates the average energy of a system to the probability distribution of its energy states. Dirichlet forms and their applications to quantum mechanics and statistical mechanics were established by [35]. Connections between Dirichlet distributions and a scale-invariant probabilistic model based on Leibniz-like pyramids are introduced by [36]. Ref. [37] showed that marginalizing the joint distribution of individual energies is a symmetric Dirichlet distribution.

### 6.4. Gene Expression Modeling

Clustering is a key data processing technique for interpreting microarray data and determining genetic networks. Hierarchical Dirichlet processes (HDP) clustering is able to capture the hierarchical elements that are common in biological data, such as gene expression data, by including a hierarchical structure into the statistical model. Ref. [38] presented a hierarchical Dirichlet process model for gene expression clustering.

### 6.5. Geometrical Probability

Thomas and Mathai [39] propose a generalized Dirichlet model application to geometrical probability problems. When the linearly independent random points in Euclidean *n* space have highly general real rectangle matrix-variate beta density, the volumes of random parallelotopes are explored. In order to evaluate statistical hypotheses, structural decomposition is provided, and random volumes are linked to generalized Dirichlet models and likelihood ratio criteria. This makes it possible to calculate percentage points of random volumes using the generalized Dirichlet marginal’s *p*-values.

### 6.6. Bayesian Analysis

Carlson’s original definition of Dirichlet averages is expressed as mixed multinomial distributions’ probability-generating functions. They also significantly contribute to the solution of elliptic integrals and have several connections to statistical applications. Ref. [40] found that several nested families are built for Bayesian inference in multinomial sampling and contingency tables that generalize the Dirichlet distributions. These distributions can be used to model populations of personal probabilities evolving under the process of inference from statistical data.

## 7. Conclusions

In this study, the fundamental ideas for the theory development of the matrix-variate Dirichlet measure in the complex domain are presented. The complex matrix-variate type-2 Dirichlet measure and averages over some useful matrix-variate functions are discussed. We establish the Dirichlet measure of the rectangular matrix-variate and the relationship between Tsallis entropy and Dirichlet averages and identify a few applications in various domains. Additionally, a few applications are covered. 

## Data Availability

No new data were created or analyzed in this study. Data sharing is not applicable to this article.
